# The Technique of Extraction

**Published:** 1900-06-15

**Authors:** Wm. Guy


### THE TECHNIQUE OF EXTRACTION.

BY WM. GUY, L.D.S.

The paper I now submit owes its inception mainly to the interest which by force of circumstances I am compelled to take in the teaching of our art and the education of our students. Many students and graduates, medical and dental, resort to my department at the Royal Infirmary, the medicals to learn *ab initio* something of extraction, the dentals to acquire by practice increased facility in the performance of an operation with which they are, presumably, already familiar. I have now had what I may venture to call a considerable experience with dental students, and from experience one gathers some definite impressions. With all these impressions I shall not weary you; the one I wish to bring prominently under your notice is the conviction that, judging from the extraordinary diversity of the methods adopted by dental students in performing this apparently simple operation, there must be a corresponding diversity in the methods of those from whom they derive their instruction. The proposition that our students of to-day are our dentists of to-morrow is sufficiently obvious, and I will not labor it; from it, however, arises the reflection that diversity of method seems likely to become as constant a character in the future as it has been in the past. I should not regard this as an unhappy augury if at the same time I thought it was likely to lead by the law of natural selection to the elimination of the useless or less useful methods, and the survival of the fittest. It, however, would imply a careful comparison by teachers and students alike, of the merits, real or alleged, of every recommended procedure, a judicial pronouncement in favor of the best, and a rigid rejection of the rest, supplemented by a careful and moderate statement of reasons. This is what is done in the case of every other surgical operation; there seems to be no reason why extraction alone should be a sort of "go as you please" affair. I make a practice of asking students why they do this, or that; the invariable reply is "Because I saw Mr. A (or Mr. B) do it." This, though one would prefer evidence of the scientific habit of mind, certainly exhibits a child-like faith on the part of the student—the pity of it is that he does not perceive that his reply is not in the least an answer to the question.

The meagre descriptions of the books are disappointing in the extreme to one who consults them with the view of comparing the directions there given. The disappointment is accentuated when one remembers the careful, minute and perspicuous directions for the performance of each and every step of an operation, which modern surgical writers find it necessary to give. A good deal, I think, might be done by our Society if members would describe and justify their own styles of operating, and if I now with all becoming diffidence submit my own methods to the crucible of criticism, I trust that others will be found ready to follow my example.

I shall describe the operation with special reference to:

(1) The Position of the Patient;
(2) The Positions of the Operator;
(3) The Conduct of the Left Hand of the Operator;
(4) The Introduction and Application of the Forceps;
(5) The Extraction;
(6) The Instruments required.

(1) *The Position of the Patient.*—The patient should be seated in the chair in as comfortable and easy a position as possible, the chair should be very slightly tilted backward, the top of the patient's head should be on a level with the operator's collarbone when he stands erect beside the chair. The head should never be thrown back and the neck stretched, especially when an anesthetic is given, as this impedes both deglutition and respiration, and also adds to the risk of blood, etc., getting into the respiratory passages. If, as is almost universal now-a-days in private practice, gas, or gas and ether is used, the gag, (I use three sizes of Hewitt's on a cord), should be adjusted and the patient directed to keep the hands in the lap, with the fingers interlocked; the patient should also be warned not to brace the feet against the foot-rest.

(2) *Positions of the Operator.*—These are three in number. I place them in the order in which I assume them when operating on both sides of both jaws.

(i.) To extract cheek teeth from the left side of the lower jaw.—I stand on the left side, the left knee slightly bent, the side of the left thigh pressing against the arm of the chair, the right foot thrown a little forward, the trunk inclining rather backwards and to the left.

(ii.) To extract the right cheek teeth and the anterior teeth from the lower jaw.—I either stand behind the patient on a kidney-shaped platform or stool, bending forward, so that I can look backwards and downwards into the mouth; or lower the chair so that I can assume a similar position standing on the floor.

(iii.) To extract teeth from the upper jaw.—I stand on the right front of the patient; both thighs are now against the arm of the chair, the poise of the head is instinctively that best adapted to secure a good view of the parts operated on.

When extracting lower cheek teeth on the left side, I never stand on the right and operate "across," because the only argument that could influence me to do so would be that it saved time, and I am convinced that if any time is saved it is inappreciable to my senses. If teeth are to be taken from both sides of the lower jaw, I am emphatically in favor of the positions I advocate, though I am willing to concede that it is admissible to operate "across" if, say, one lower molar left and one or more upper teeth require extraction.

(3) *The Conduct of the Left Hand.*—This is the point in the technique of extraction on which I am disposed to lay the greatest stress, because nowhere have I been able to find coherent or reasonable instruction thereanent. The principle on which I take my stand is this: That in every case the alveolar borders at the point where the forceps are to be applied should be firmly grasped between the forefinger and thumb of the left hand. In comparison with this, the use of the fore and middle fingers, or the pushing up of the lip with the thumb, appear to me impotent and unworkmanlike.

Taking first the left lower cheek teeth: The thumb on the buccal side slightly crooked keeps away the cheek, the forefinger the tongue, the remaining fingers pressed under the jaw grip and support it. In operating on the right lower cheek teeth, the left arm surrounds the patient's head, the forefinger is now on the buccal, the thumb on the lingual side of the alveolar margin, the remaining fingers under the jaw. In this way complete control of the lower jaw is obtained. For the anterior lower teeth the forefinger is on the labial side, the thumb on the lingual.

For cheek teeth in the upper jaw on the left, the forefinger is on the buccal, the thumb on the palatal side.

For upper front teeth the forefinger is on the labial, the thumb on the palatal side. The thumb here I always keep slightly bent; this affords a shelf or inclined plane which directs teeth or roots that "jump," or slip forward, out of the mouth, instead of into the larynx.

For upper right cheek teeth the wrist is bent, the elbow raised, the thumb is on the buccal side, the forefinger on the palatal.

In introducing the finger and thumb to the back of the mouth, they should be carried well back first, and then opened to grasp the alveolar margins.

(4) *Introduction and Application of the Forceps.*—In the case of the cheek teeth the blades of the forceps should always be entered in a plane parallel to the occlusal surfaces of the teeth, and so carried to the tooth it is desired to extract, when, for upper teeth, a slight depression of the hand, for lower teeth, a turn of the wrist, enables one to instantly and correctly apply them—a process in which the finger and thumb of the left hand will be found to greatly assist one's sense of accurate adaptation. To the front teeth, above and below, the blades are advanced in a plane parallel to the long axis of the tooth.

(5) *Extraction.*—The forceps should first be driven well home, but this should be done "in once"; nothing annoys me more than to see a student prod, prod, prodding at a tooth with his forceps, unless it is to see him start to "rock" it. In the case of conical roots this driving home of the blades often evicts them at once, the principle involved being the well-known one that two bodies can not occupy the same space at the same time. But as a rule, the tooth being now grasped well below the neck, some extractive force has to be applied.

Take first the upper cheek teeth. The force is applied in the outward direction at once. I never apply the force inwards to begin with, as is always advised. I regard it as a waste of time and energy. I am in the habit of drawing the students' attention to the fact that we do not "pull" teeth, and that the dislodging force is mainly supplied by the operator bringing his weight to bear on the forceps, rather than by the exercise of any great muscular effort. The jaw, firmly grasped by finger and thumb, is kept steady and is easily controlled; the finger and thumb, too, tell when a tooth is yielding, prevent more than the minimum amount of bending or giving of the outer alveolar plate, and also control hemorrhage.

To the lower cheek teeth the force is also applied in a direction which is mainly outwards, though also upwards and slightly forwards.

I assume that hawksbill forceps are used, when extraction is achieved by depressing the handles. The main factor here is the weight of the operator controlled by the muscles of the trunk and shoulder girdle.

The upper front teeth, when grasped firmly, are rotated, the effective muscles here being the supinators.

In the case of the lower front teeth, they are as a rule very easily lifted from their sockets by depressing the handles of the forceps (hawksbill). I stand behind the patient for these teeth. While depressing the handles it will be found that the supinators are again brought into play, but no rotation is attempted or effected.

(6) *Instruments employed.*—I use only two pairs of forceps—one for the lower, one for the upper jaw. They are somewhat like the root forceps, Nos. 74 and 30, in Ash's Catalogue, but are modified to meet my views The blades are somewhat stouter and a trifle longer than usual, the curve of the blades in the uppers is a little more decided. In both, the points of the blades are fairly broad, and must meet when closed. I am quite prepared to be told that these two pairs are insufficient, but "*experientia docet,*" I have to extract between 5,000 and 6 000 teeth annually at the Royal Infirmary alone. I never allow the students to use any other forceps, and it certainly does not happen more than once in twelve months that any others are required.

I use elevators. However, I never want them in the upper jaw, and in the lower jaw I find them mainly useful for turning out in succession a number of molar and bicuspid roots. For this end I enter the elevator on the distal side of the distal root and work forwards, so that rotation, depression, and carrying backwards of the handle keep the point directed toward the front of the mouth. Of course there are exceptions to this, as when the mesial root of a second molar may be dislodged by entering the elevator between it and the up-standing first molar. But I discourage the use of the elevator with the blade looking backwards, and I never use or permit any student to use a straight elevator, an instrument which in my opinion should be relegated to repose in the Museum alongside the key.

### DISCUSSION.

Mr. J. S. Amoore, L.D.S., was very much indebted to Dr. Guy for having laid down his views so clearly and explicitly. This was a matter upon which they had each of them considerable experience, and there would be no difficulty in discovering points of difference, and there was more than one in which his ideas ran directly counter to those of Dr. Guy. Firstly, he had pronounced his opinion that the instruments should be confined to two pairs of forceps, an upper and a lower pair of stumps, and a pair of right and left elevators to the exclusion of the straight elevator, against which he had inveighed in no qualified terms. Now he would be the last to question that in skilful hands this armature might leave little to be desired, but in less skilful hands and with beginners he deprecated it entirely. He (Mr. Amoore) was not one of those who thought that the best results were obtained by the use of the fewest instruments (unless the attainment of speed was the paramount question); a pair of root forceps was not, in his opinion, the best instrument for removing a strongly implanted molar, nor a curved pair of stump forceps the ideal one for a beginner to extract a central with, and he failed to appreciate Dr. Guy's aversion to the straight elevator, which for lower wisdoms was, in his opinion, almost indispensable.

There was another point in which he differed from Dr. Guy, and that was the position he took up in standing on the left side of the chair to remove lower teeth with the hawksbill forceps, and he thought it an inconvenient method, as it necessitated the operator changing his position if removing teeth from both sides of the mouth, and, if under "gas," involved some loss of time, a disadvantage which Dr. Guy had admitted, and in most of their consulting rooms the left side of the chair was encumbered with a spittoon (often a more or less complicated one) and a bracket. And when the operation could be as successfully performed from the right hand side of the chair, it seemed to him inadvisable to teach students a method which was attended with certain disadvantages. He thought, too, that Dr. Guy placed his patient rather high in relation to the operator for the convenience of the latter. If he rightly understood him, he considered that the patient's head should be on a level with the operator's shoulder. In the case of the lower teeth he obviated this disadvantage by standing on a raised platform or stool behind the patient, but even with the upper teeth he thought that in this position there was some loss of power.—*Dental Record.*

